# Learning Doctor-Patient Communication – Evaluating the effectiveness of the communication training course at Leipzig University from the students' point of view

**DOI:** 10.3205/zma001042

**Published:** 2016-05-17

**Authors:** Jana Cämmerer, Olaf Martin, Katrin Rockenbauch

**Affiliations:** 1 University of Leipzig, Faculty of Medicine, Department of Medical Psychology and Medical Sociology, Leipzig, Germany; 2 Martin-Luther-University Halle-Wittenberg, Institute of Medical Sociology, Halle (Saale), Germany

**Keywords:** doctor-patient communication, training in medical school, learning objectives, communication curriculum, communication skills

## Abstract

**Objective: **At the University of Leipzig, the requirements of the Licensing Regulations for Doctors (Approbationsordnung für Ärzte) for the practical training of communication skills are actively implemented by a two-semester communication course. During this course, student tutors impart the basics of interpersonal as well as selected aspects of doctor-patient communication using interactive training methods. This article reports on the effect the training has on the self-assessed communication skills of the medicine students.

**Methods: **The students’ self-perceived communication skills were assessed, both at the beginning and after the completion of the first and second course semesters using questionnaires related to the course’s learning goals. Pre-post comparisons were then carried out. 142 students (of 163 students in total) participated in the survey at the start of the course, of which 117 completed the T2-questionnaire at the end of the first course semester. Only the 84 students who also completed the questionnaires in the second course semester were included in the statistical analysis. These responses were analysed using both descriptive and inferential statistics.

**Results: **The comparison of the self-assessments between the four measurement points showed that statistically significant learning progress for all assessed communication skills had taken place from the point of view of the students. The largest changes between measurements, and therefore the greatest learning progress, could be seen in knowledge related skills.

**Conclusion: **From the students’ point of view the communication training contributes significantly to the acquisition of communication skills. The results suggest that this “hands-on” course concept is suited to successfully enhance the students’ communication skills. The course concept should therefore be retained for both the course in its current form as well as for any extension of the course into the clinical part of medical school. However, further assessments on the exam results and long-term effects should take place.

## Zusammenfassung

**Zielsetzung:** An der Universität Leipzig werden die Forderungen der Approbationsordnung für Ärzte nach praxisnahem, Kommunikationskompetenzen förderndem Unterricht u.a. durch einen zweisemestrigen Gesprächsführungskurs aktiv umgesetzt. In diesem Kurs vermitteln studentische Tutoren mit Hilfe anwendungsorientierter Methoden die Grundlagen zwischenmenschlicher Kommunikation und ausgewählte Aspekte ärztlicher Gesprächsführung. Dieser Beitrag berichtet darüber, welchen Effekt der Gesprächsführungskurs auf die selbsteingeschätzten Gesprächsführungskompetenzen der Medizinstudierenden hat.

**Methode:** Mittels lernzielspezifischer Fragebögen wurden zu Beginn und nach Abschluss des ersten und zweiten Kurssemesters die selbsteingeschätzten Gesprächsführungskompetenzen der Studierenden erhoben und Vorher- Nachher-Vergleiche der Einschätzungen durchgeführt. An der Fragebogenerhebung nahmen zu Kursbeginn 142 Studierende (von insgesamt 163 Kursteilnehmern) teil, von denen 117 auch den T2-Fragebogen am Ende des ersten Kurssemesters und 84 Studierende die Fragebögen im zweiten Kurssemester ausfüllten und damit in die statistische Auswertung eingingen. Die Fragebögen wurden mittels deskriptiver und inferenzstatistischer Methoden ausgewertet.

**Ergebnisse: **Der Vergleich der Selbsteinschätzungen zu den vier Messzeitpunkten ergab bezüglich aller erhobenen Gesprächsführungskompetenzen statistisch signifikante subjektive Lernfortschritte. Die größten Differenzen zwischen den Messzeitpunkten und damit die größten Lernfortschritte ergaben sich für wissensbezogene Kompetenzen.

**Schlussfolgerung: **Der Gesprächsführungskurs trägt aus Studierendensicht bedeutsam zur Vermittlung kommunikativer Kompetenzen bei. Die Ergebnisse sprechen dafür, dass durch das praxisorientierte Kurskonzept die Gesprächsführungskompetenzen der Studierenden erfolgreich erweitert werden können. Für die Weiterführung des Kurses und eine empfohlene Ausweitung auf den klinischen Teil sollte das Kurskonzept daher beibehalten werden. Es sollten jedoch weitere Evaluationen zu Prüfungsergebnissen und Langzeiteffekten erfolgen.

## Introduction

Due to the growing awareness of the great influence of doctors’ social and communication-related skills on the interaction with patients as well as on the successful treatment outcome [1], [2], [3], [4], [5], [6], [7], [8], the teaching of communication skills during medical training has gained in importance in recent years. 

It is therefore important to note that the training of those skills now forms part of the Licensing Regulations for Doctors (Approbationsordnung für Ärzte) [http://www.gesetze-im-internet.de/_appro_2002/BJNR240500002.html], which states that doctor-patient communication skills are to be taught and assessed during medical training.

The University of Leipzig’s Faculty of Medicine fulfils these requirements, inter alia, through a communication skills training course in the third and fourth semester of medical school.

During this course, which has been offered since winter semester 2003/2004, the basics of interpersonal as well as selected aspects of doctor-patient communication are taught [9]. 

**Figure 1:**
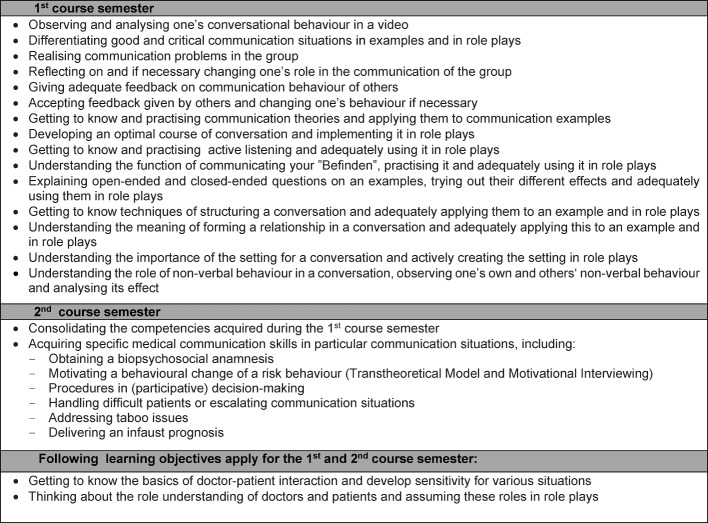
Overview of the learning objectives in the first and second course semester

The mandatory course spans two semesters with a total of 39 teaching hours. It is carried out in groups of 8-12 students and lead by student tutors [10], [11]. The main objective of the course is to impress upon the students the importance of a patient-oriented attitude and a situationally appropriate, empathetic conversational style and to give the opportunity for these skills to be practiced. To achieve this, specific cognitive, affective and behavioural learning objectives are set for each semester, which provide a rough framework for required topics to be addressed (see Figure 1 (Fig. 1)). These learning objectives include teaching content, which is explicitly discussed and consciously trained, as well as more implicit knowledge and skills, which emerge from the experience gained during the course.

The entire course concept is aimed at the students practically applying the acquired knowledge and skills during the course. By working in small groups of 3-4 people a motivating atmosphere, conducive to learning, is generated [12], [13]. The transfer of theoretical knowledge into practical skills is facilitated by a wide range of role-play and practical exercises [14]. Students are then offered the chance to reflect on their conversational behaviour through feedback and video analysis of these exercises and are motivated to alter and improve it [15], [16]. Role-play is of particular importance, since it allows the students to experience realistic communication situations in a controlled environment. The students have the opportunity to try different roles to practically apply the communication techniques that have been taught in a defined sample situation [17]. After the role-play the students receive feedback on their conversational behaviour. This feedback forms a vital part of the learning success throughout the course for both the active participant (doctor role) and the students giving feedback. The passive roles (e.g. the patient role) are largely played by fellow group members. In the second course semester some of the passive roles are played by professional actors [18]. Using these simulated patients allows a significantly more realistic and complex conversation situation to be simulated than would be possible with fellow students [19]. In addition to evaluating the role play through group feedback, a video analysis based on the recorded conversation is usually carried out. Predetermined observation tasks related to communication methods are discussed using this video analysis. This allows concrete examples from the conversation to be used to reinforce the key teaching points. 

The course finishes after the second course semester with a video-based exam, that tests to what extent the students’ can apply what they have learned to a practical example. In addition, the training course is evaluated by the students at the end of each course semester. However, neither the exam nor the students’ feedback provide any information on the knowledge and abilities the students already had before starting the course and which competencies have been acquired through active participation in the course. Furthermore, nothing can be said, as to how the students themselves assess the impact the course has on their communication skills.

The present study was conducted with the aim to close this gap. 

## Methods

The present study is a quasi-experimental study. The medical students were asked to assess their own knowledge and ability to apply specific communication skills by completing a questionnaire at four specific points of the course. The changes in these abilities due to the students’ participation in the Leipzig communication course were examined in this study.

### Questionnaires

The questionnaires for this study were specifically developed based on the course’s learning objectives mentioned above. They were aimed at determining how competent the students feel about their own communication skills. 

**Figure 2:**
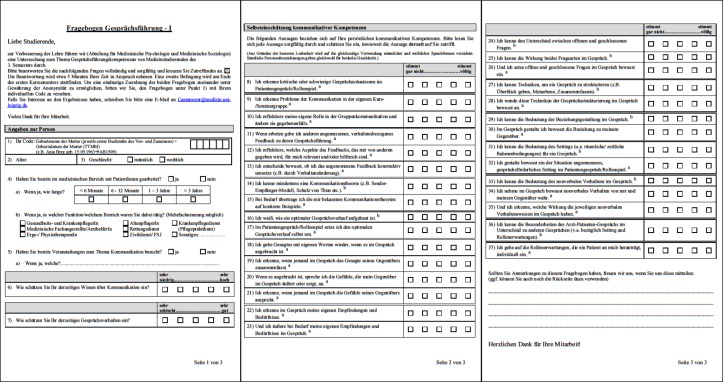
T1-questionnaire (^a^ skill-related items; ^b^ knowledge-related items).

The T1-questionnaire (see Figure 2 (Fig. 2)) consists of three sections with a total of 37 items. In section one the students’ sociodemographic data (age, gender, pre-existing experience in working with patients and previous participation in communication trainings) was assessed. In section two the students were asked to assess their self-perceived communication knowledge and conversational behaviour in general whereas in section three they had to assess their specific competencies related to the course’s learning objectives. The self-assessment of the competencies related to the learning objectives consisted of 30 items, of which 9 items assess the students’ knowledge regarding the course’s learning objectives (knowledge related = "to know how") and 21 assess the students’ confidence applying these skills and recognising relevant behaviours and issues (skill-related = "to show how") [20], [21]. 

These self-report measures of competence required the students to assess their level of agreement to a series of self-referred statements on a 5-point Likert scale.

The T2-questionnaire revisits all questions of the T1-questionnaire for pre-post comparison - with the exception of the two items in the T1-questionnaire which assessed the students’ previous practical and theoretical experience.

**Figure 3:**
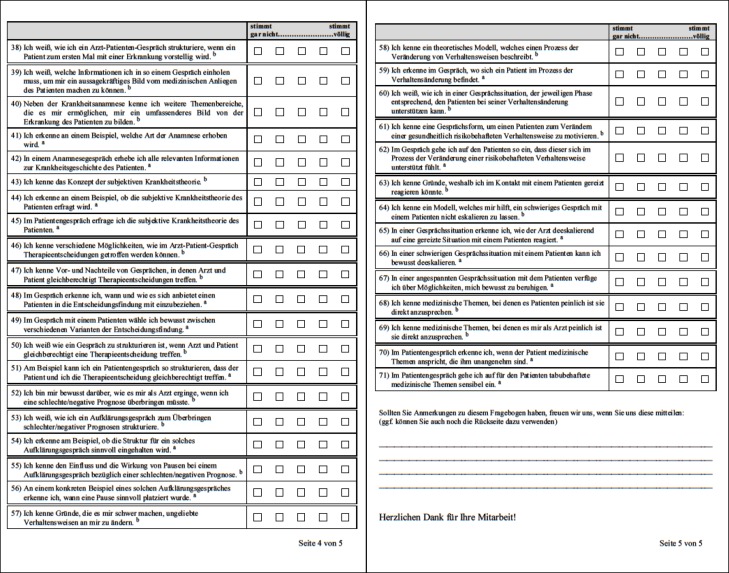
Additional items of the T3/T4-questionnaire (^a^ skill-related items; ^b^ knowledge-related items).

For the measurements at T3 and T4, 34 statements relating to the contents of the second course semester were added to the T1/T2-questionnaire (see Figure 3 (Fig. 3)). These 34 items can again be subdivided into 18 items assessing the students’ knowledge ("to know how") and 16 items assessing the students’ practical skills (“to recognise", "to be able to" and "to do" = "to show how").

A confirmatory factor analysis, which was conducted for statistical confirmation of the division of the items into the subscales "to know how" and "to show how", revealed no meaningful factors. However, the reliability analysis showed an acceptable degree of internal consistency for both scales with a Cronbach's alpha of α=.78 for "to know how" and α=.75 for "to show how".

#### Recruitment and Sample 

Of the 325 students participating in the course during the survey period, those 163 students who completed the course in the second half of the first semester were surveyed in this study.

The T1-questionnaire was handed out to the course participants at the beginning of the first course unit. It was completed by 142 students, of which 117 students also completed the T2-questionnaire at the end of the first course semester. The T3-questionnaire that was issued at the beginning of the second course semester was completed by 90 students. The T4-questionnaire that was given out at the end of the second semester was completed by 88 students. Only those 84 students who provided data at each of the four measurement points were included in the statistical analysis. Subsequently, only these complete data sets are discussed in the following report. 

#### Data Analysis

First, descriptive statistics of the sample (age, gender and existing experience) and all dependent variables (communication knowledge, conversational behaviour, to know how_1, to show how_1, to know how_2, to show how_2) were conducted. Second, the influence of the demographic factors (factor levels see Table 1 (Tab. 1)) on the dependent variables was evaluated using multivariate analysis of variance (MANOVA) for cross-sectional questions and repeated measures ANOVA was used for longitudinal questions with Bonferroni's post hoc test for multiple comparisons. Differences between the competence dimensions (knowledge vs. behaviour or know vs. show) were analysed using paired samples t-tests. Using repeated measures ANOVA with time (measurement point) as the within-subjects factor, the learning progress over all four measurement points was examined. The Bonferroni correction was used as a post hoc test for multiple comparisons. All analyses were performed using SPSS (Statistical Package for Social Sciences) version 18.

For ease of interpretation and comparison of the 30/64 items related to the learning objectives, the variables “to know how_1” and "to show how_1" (for the learning objectives of the first course semester, measured at T1, T2, T3 and T4) and the variables "to know how_2 "and" to show how_2" (for the learning objectives of the second course semester, measured at T3 and T4) were generated. This subdivision of variables is based on the previously described classification into "knowledge-related" and "skill-related". These new variables result from the means of the items belonging to the respective category. A subdivision of the skill-related variable "to show how" into the single variables "to recognise", "to be able to" and "to do” was not necessary, since the individual analysis of these variables resulted in no change to the overall results.

## Results

### Description of the Sample

Table 1 (Tab. 1) shows the socio-demographic characteristics of the sample at T1. 52 (62%) female and 32 (38%) male students aged between 18 to 30 years took part in the survey. The mean age is 21.9 years (SD=2.6). This distribution roughly corresponds to the gender distribution in the entire second academic year (61%:39%) (Teaching Unit, personal information, 01.03.2012.). About half of the respondents (45%) had already worked with patients outside the Patient Care Traineeship (Pflegepraktikum). 51% had only gained experience dealing with patients in the course of the Patient Care Traineeship. Here, a significant correlation between the age of the students and their experience with patients can be observed (r_s_=.49, p<.001). 90% of the students over 25 years old stated that they had worked with patients outside the Patient Care Traineeship. In the age group of 21-24 years old, this dropped to only 46% of the respondents and in the group of 18-20 years old to only 19%. In both age groups, the students had dealt with patients mostly during Patient Care Traineeship. The duration of this work increases with age, too. Overall, 20% of the respondents had already participated in communication-related courses, such as seminars on communication, mediation and dispute/conflict resolution before.

#### Self-assessment of skills

As shown in table 2 (Tab. 2), the means of the knowledge-related variables "communication knowledge" (Item 6) and "to know how_1" at the first measurement (T1) are lower than those of the skill-related variables "conversational behaviour" (Item 7) and "to show how_1 "(t(83)=-4.03, p<.001 or t(83)=-4.22, p<.001). At T2, this effect is reversed and the self-assessed knowledge shows higher mean values than the self-assessed skills (t(83)=3.16, p<.002 or t(83)=14.78, p<.001). These higher mean values of the knowledge-variables compared to the skill-variables continue for T3 (t(83)=2.21, p=.03 or t(83)=15:15, p<.001) and T4 (t(83)=5.6, p<.001 or t(83)=12.29, p<.001). This trend can also be observed for the additional self-assessments regarding the learning objectives of the second course semester at the two measurement points (T3:ns/T4:t(83)=4:36, p<.001).

**Figure 4:**
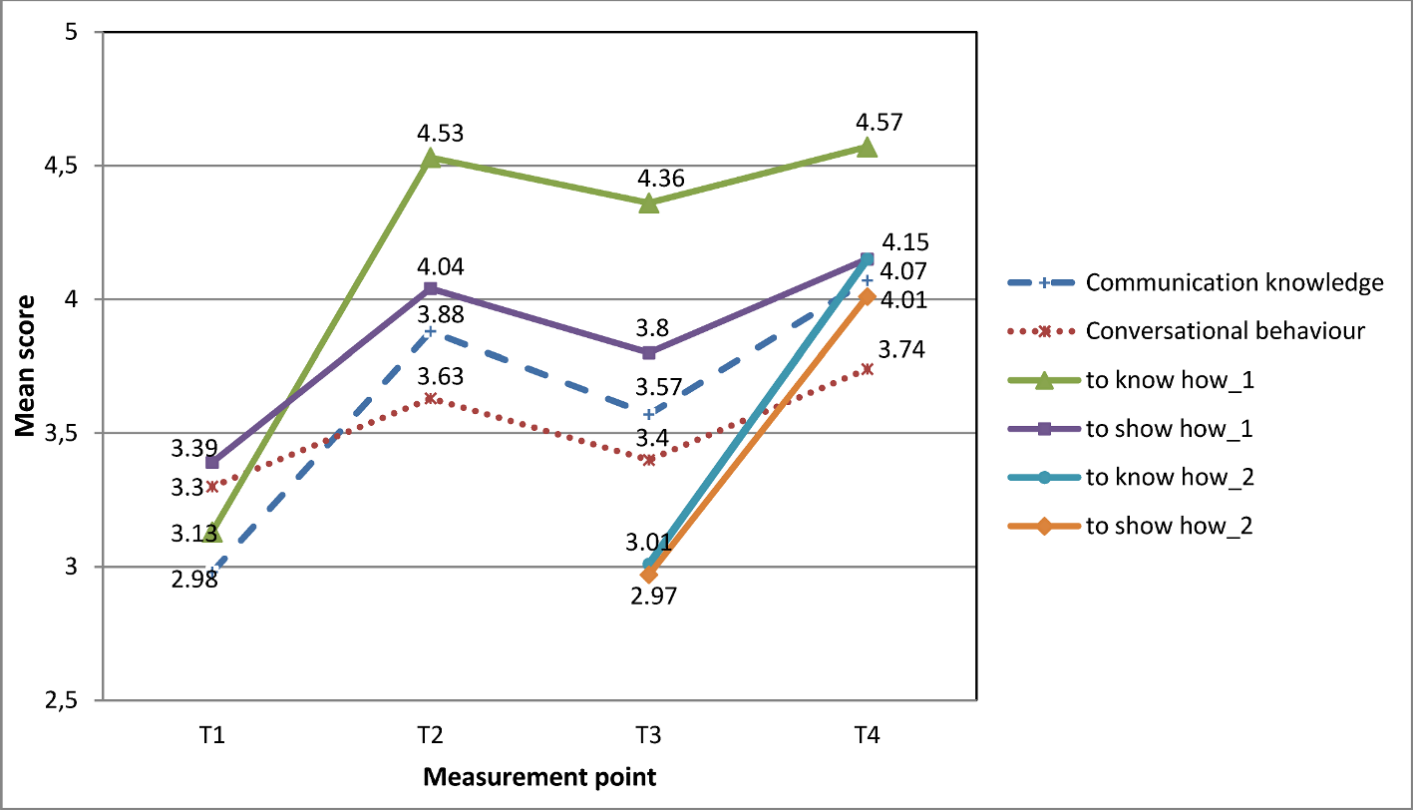
Mean-scores of the student’s self-assessments at T1, T2, T3 and T4

**Figure 5:**
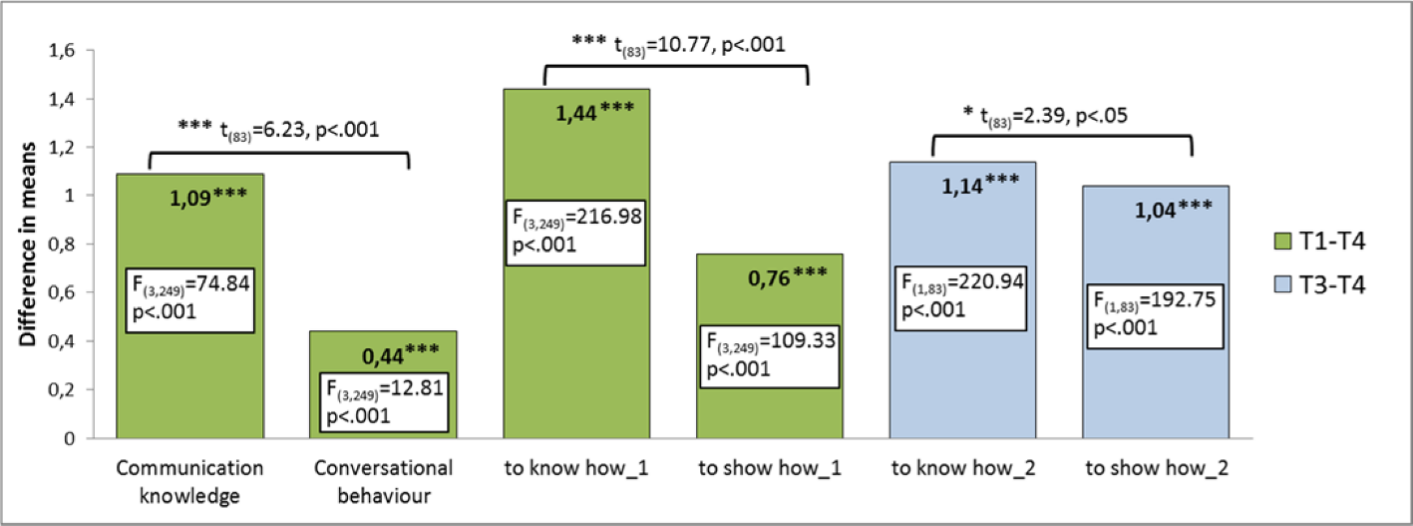
Learning progress over the course (comparison of T1-T4 & T3-T4)

The comparisons between the four measurements (see Figure 4 (Fig. 4)) show that the mean values of all variables assessed at T1 increase significantly from T1 to T2, decrease significantly at T3 (but remaining above base level (T1)) and increase significantly at T4 to a higher level than at T2 (for F and P values see table 2 (Tab. 2)). As illustrated in figure 5 (Fig. 5), highly significant increases in the mean values of all variables can be observed over the two course semesters. Regarding the competence dimensions, in the T1-T4 comparison the significantly higher increases exist consistently for the knowledge-related variables (t(83)=6.23, p<.001 or t(83)=10.77, p<.001).

The variables solely related to the learning objectives of the second course semester also show a significant increase in the mean values from T3 to T4, which is significantly higher for both variables than for those of the first semester (t(83)=-12.45, p<.001 or t(83)=-9.38, p<.001). Here too, the significantly higher rate of increase exists for the knowledge-related variable (t(83)=2.39, p=.019).

Regarding the sociodemographic variables, no significant effects were found on the dependent variables or their changes over the two semesters.

## Discussion

The reported results suggest that from the students' perspective significant learning progress had taken place over the two course semesters in all the assessed competencies. The progression analysis shows significant subjective increases in all competencies after participating in the first course semester. After the semester break, although the students assessed their skills to be significantly worse than in the previous assessment those skills were still slightly higher than at the beginning of the course. After the completion of the second course semester, significant subjective learning progress can be identified for all assessed communication skills in comparison to the beginning of the semester. The competencies exclusively referring to the second course semester’s learning objectives show distinctly greater increase than the first course semester’s. Overall, the highest self-assessments at T2, T3 and T4 as well as the largest subjective learning progressions over the course were found for the knowledge-related competencies.

In order to ensure an adequate discussion of these results, some limitations of the study, especially methodological limitations, need to be discussed. First, it has to be noted that this study is a quasi-experimental one-group longitudinal study with four measurements. A randomized comparison with a non-intervention control group was not possible in this study. This kind of procedure is not uncommon in such studies [20], [22], [23], since the pre-post comparison of the self-assessed competencies allows a good insight into the students’ subjective increase in competence.

Second, the assessment of the skills is based on the students’ self-assessments and is therefore more likely to be affected by systematic measurement errors than objective performance measurements in the form of performance tests would be [24], [25], [26]. However, as shown in several studies, the self-assessment of a competence is a good predictor for that ability being applied in a real situation [27], [28], [29], [30].

Third, it should be noted that since a bespoke questionnaire was used, a comparison of the results of this study with those of other studies has limitations. In addition, factor analysis could not confirm the difference between the two assumed factors ("to know how", "to show how"), which reduces the explanatory power of these two factors.

Fourth, there is a risk of distortion in the results due to a possible tendency for socially desirable responding or due to a non-response bias resulting from distinct differences between the students that continued to respond to the survey and those who dropped out. 

To check for external influences on the results, as many relevant sociodemographic factors as possible were included. However, causal conclusions based on the results should be approached with some caution since the reported subjective increase in competence could be induced by a general increase of knowledge and skills during medical school.

Overall, the students assess themselves as being more competent regarding their communicative knowledge and skills after participating in the course. Therefore it can be concluded that the various aspects of both general and doctor-patient communication covered by the course were successfully imparted to the students. This in turn suggests that the teaching concept fulfils the didactic requirements to enhance the students’ communicational skills and to enable them to better cope with communication situations in everyday life and in medical contexts. At best, this should qualify them to manage the complex requirements of communicating with patients that are to be expected when practising medicine [31]. Thus, the findings of this study are in line with the findings of similar studies [20], [22], [23], [32]. The students’ self-assessed learning progress after completing the communication course also suggests that there is a need for more general and doctor-patient communication training at the start of the second year of study.

It is also worth considering the cross-sectional and longitudinal differences in the two observed dimensions of competency. At the beginning of the course, the students assessed their skill-related competencies considerably higher than their knowledge-related competencies. This changed after the first semester. For the three subsequent measurement points the results always showed higher self-ratings for the knowledge-related competencies, which also show the greater subjective learning progress throughout the course. In this respect, there are similarities to the results of the study by Lengerke et al. [20]].

From the students’ point of view the communication course contributes more to the transfer of knowledge than to the transfer of practical skills. This finding allows for different interpretations. One explanation could be that the medical students actually acquired more communication knowledge than practical communication skills during the course. This could be due to the course’s teaching methods themselves, which might favour knowledge acquisition ahead of the development of practical skills. Since great importance is attached to practical learning in the course and the students are trained to actively implementing what they have learned in role plays, this is unlikely. Another possibility would be that the students can not completely transfer their theoretical knowledge into practical skills regardless of the course didactics. This so-called "inert knowledge" [33]] would mean that students believe they have communication knowledge but are unable to use it effectively in realistic situations. A third alternative is that the pre-clinical part of medical school, during which this communication course takes place, has a stronger focus on teaching and testing medical expertise, as opposed to practical skills [http://www.zv.uni-leipzig.de/studium/angebot/studiengaenge/studiendetail.html?ifab_id=281]. This can result in the students’ learning focus being directed to accumulating knowledge instead of actually applying it. Another explanation would be that the reported differences are not due to the extent that the knowledge and skills have been acquired, but simply reflect the different awareness and explicability of the various learning objectives. Immediately after the course, the students are probably more aware of the acquired knowledge than of the behavioural changes which have been achieved. This may explain why it is easier to assess the increase in knowledge than improvements in their communication behaviour. Moreover, knowledge can be used and communicated more easily, whereas new behaviour must develop over time through practice. It can therefore be assumed that through a more frequent application of the conveyed skills, the assessments of knowledge and actual behaviour will slowly align. 

It is also possible, that by participating in the course the students have become more aware of the difficulty of practically implementing communication knowledge into concrete behaviour. Therefore they might be able to assess their skills in a more critical (and perhaps more realistic) way. 

## Conclusions

The reported self-perceived learning progress suggests that the students are interested in communication-related issues and willing to learn about them. It is also necessary to attach greater importance to communication skills not only during medical school but also in medicine in general. 

Communication competence must be regarded as one of the key skills in the medical profession [34] and its advancement should therefore be firmly anchored in the medical curriculum. Amongst the approaches for realizing this long-term goal are the recently adopted National Competence Based Catalogue of Learning Objectives for Undergraduate Medical Education (NKLM) [http://www.nklm.de/] and other recommendations on learning objectives for medical curriculums [35], [36].

It should also be noted that a survey of students’ communicative knowledge and skills over only two course semesters only allows a snap-shot of their communication skills. The targeted long-term effect of the training should therefore continue to be researched in a longitudinal study.

Regarding further research, it would be very instructive to perform a questionnaire survey with an additional control group of medicine students, who did not attend a communication course or any similar training. This would give more information on possible external factors as well as offer a better understanding of the actual effect of the course. 

As described earlier, the students were required to take an exam after the second course semester. The knowledge and skills gained during the course have to be applied to pass this exam. By comparing the self-assessed communication skill levels with the individual test results, the extent to which the subjective learning outcomes match the test performance could be tested. In addition, the students’ subjective self-assessment could be compared to the objective performance data. Moreover, a more detailed insight into the effects of the course would be possible and the course could be optimised to the needs of the participants using such a procedure. In addition, a comparison of the self-assessed skills with the course evaluation results could be used to evaluate whether the individual course evaluation has an impact on the subjective learning progress and / or the exam results. Overall, the combination of self-assessment, examination and evaluation could provide a very differentiated picture of how students perceive the communication course and its learning effects as well as how these perceptions are related to the objective performance data. The development and implementation of an Objective Structured Clinical Examination (OSCE) would also be desirable for the future, since this exam format allows a practical and objective assessment of skill-related knowledge and practical clinical competence [37]. 

In summary, the problem- and application-oriented, practical course concept and its methodological implementation can be judged as effective (from the students’ point of view) and successful. The course should therefore be continued in its current form. As this study has also shown self-assessed communicative knowledge and skills decrease if the students do not deal with these issues on a regular basis. This communication curriculum should therefore be continued during the clinical part of medical education. This would shorten the lag between learning and applying the acquired knowledge and skills in the interaction with real patients and would enable a closer link between theory and practice. The continuation of this training would give future physicians the opportunity to deal with communication-related and psychosocial issues over the entire course of their medical training and therefore gain a wealth of practical experience. This, in turn, would aid retention of what has been learned during the course and its transfer into professional practice.

## Competing interests

The authors declare that they have no competing interests.

## Figures and Tables

**Table 1:**
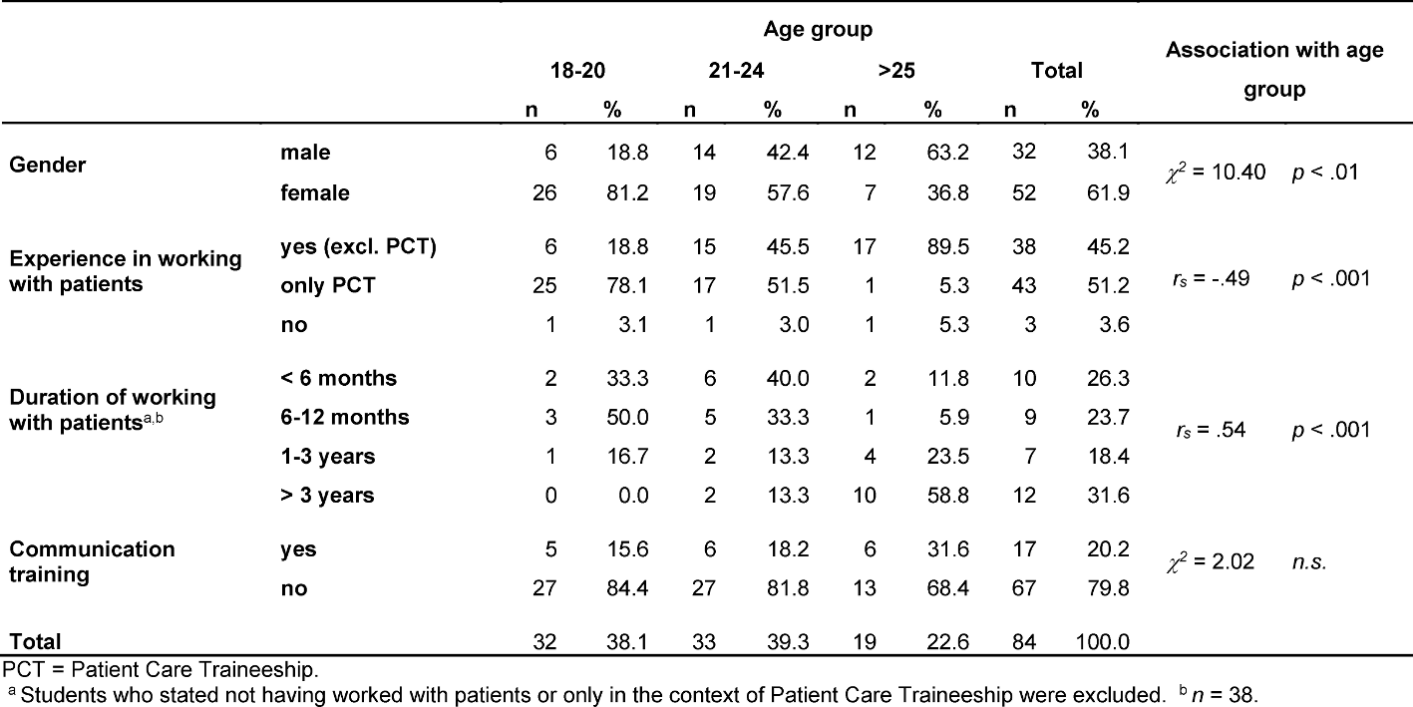
Descriptive statistics of the sample (N=84)

**Table 2:**
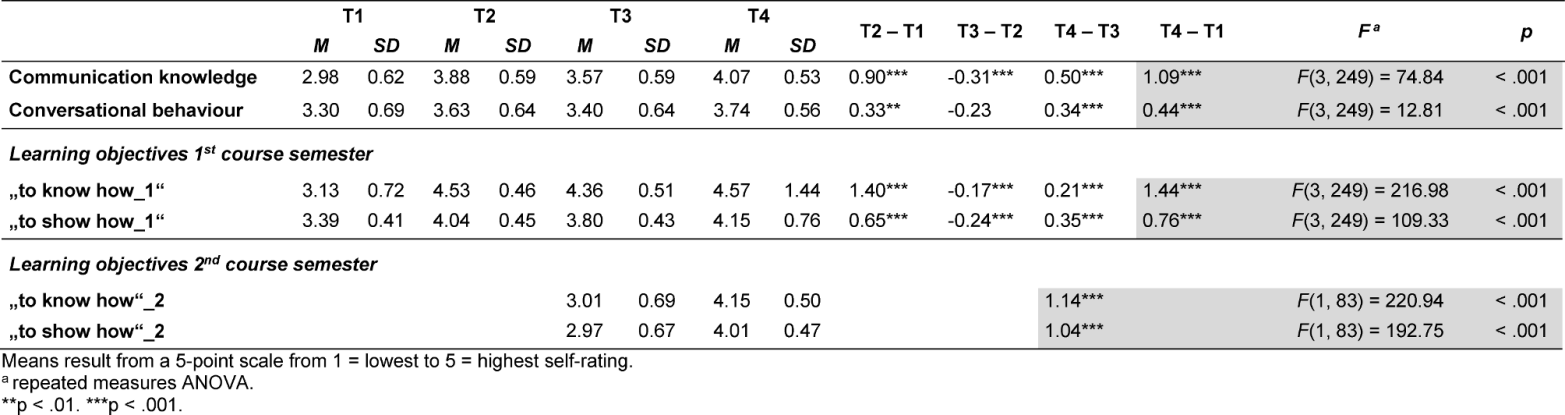
Descriptive and inferential statistics of study variables (N=84)
